# Rare case of circumferential esophageal peeling

**DOI:** 10.1002/ccr3.2846

**Published:** 2020-04-13

**Authors:** Kentaro Tominaga, Atsunori Tsuchiya, Hiroki Sato, Yui Ishii, Nobutaka Takeda, Kazuki Natsui, Yuzo Kawata, Naruhiro Kimura, Yoshihisa Arao, Suguru Takeuchi, Kazunao Hayashi, Junji Yokoyama, Shuji Terai

**Affiliations:** ^1^ Division of Gastroenterology and Hepatology Graduate School of Medical and Dental Sciences Niigata University Niigata Japan

**Keywords:** circumferential esophageal peeling, foreign body, Nikolsky phenomenon, pemphigus vulgaris

## Abstract

This report highlights the easy peeling of the esophageal epithelium with Nikolsky phenomenon after swallowing the foreign body and the healing status of the esophagus only 3 days later in a patient of pemphigus vulgaris.

## Graphical abstract

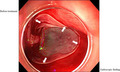

## 1 QUESTION

**FIGURE 1:**
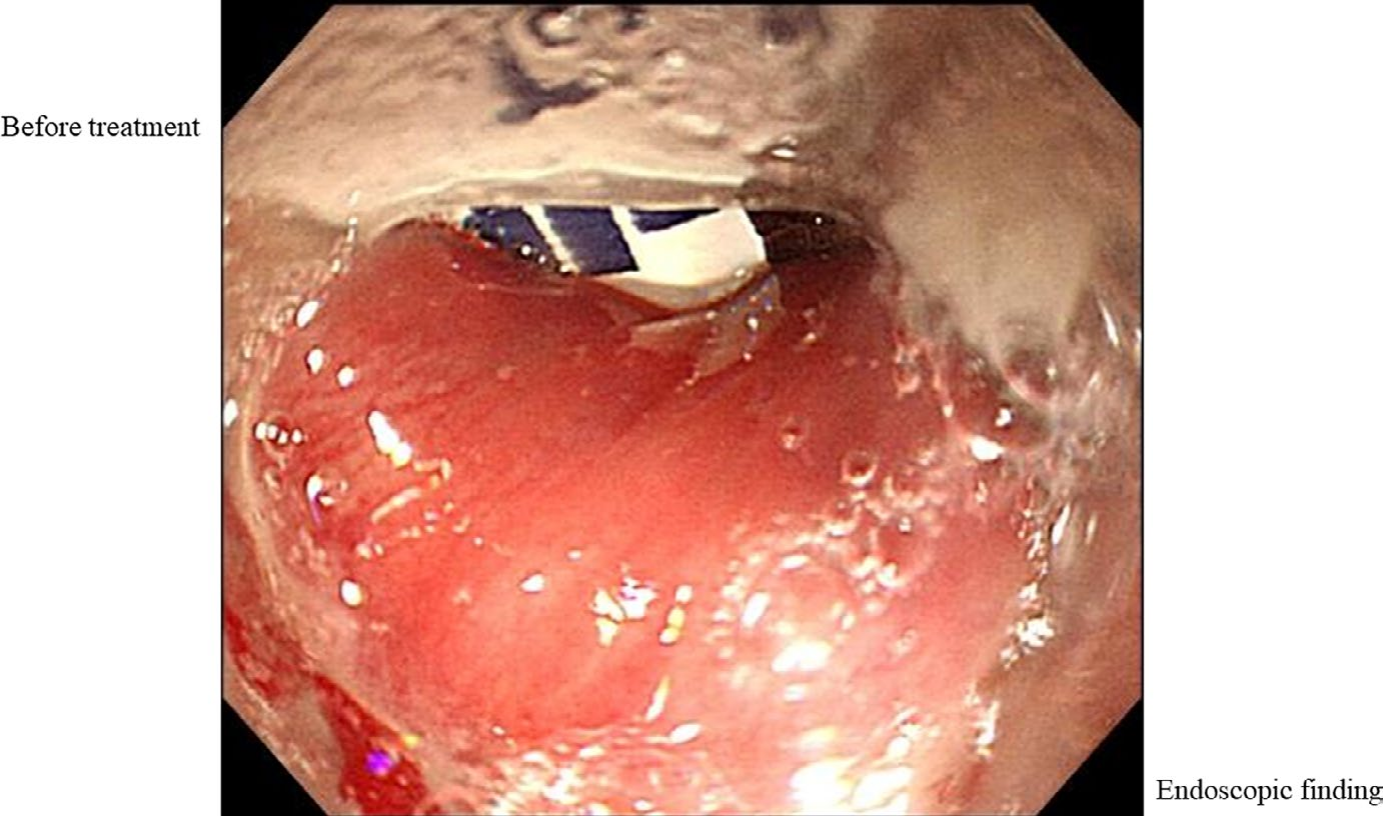
Upper gastrointestinal endoscopy shows a stuck press through package (PTP) in the pharyngoesophageal junction

**FIGURE 2:**
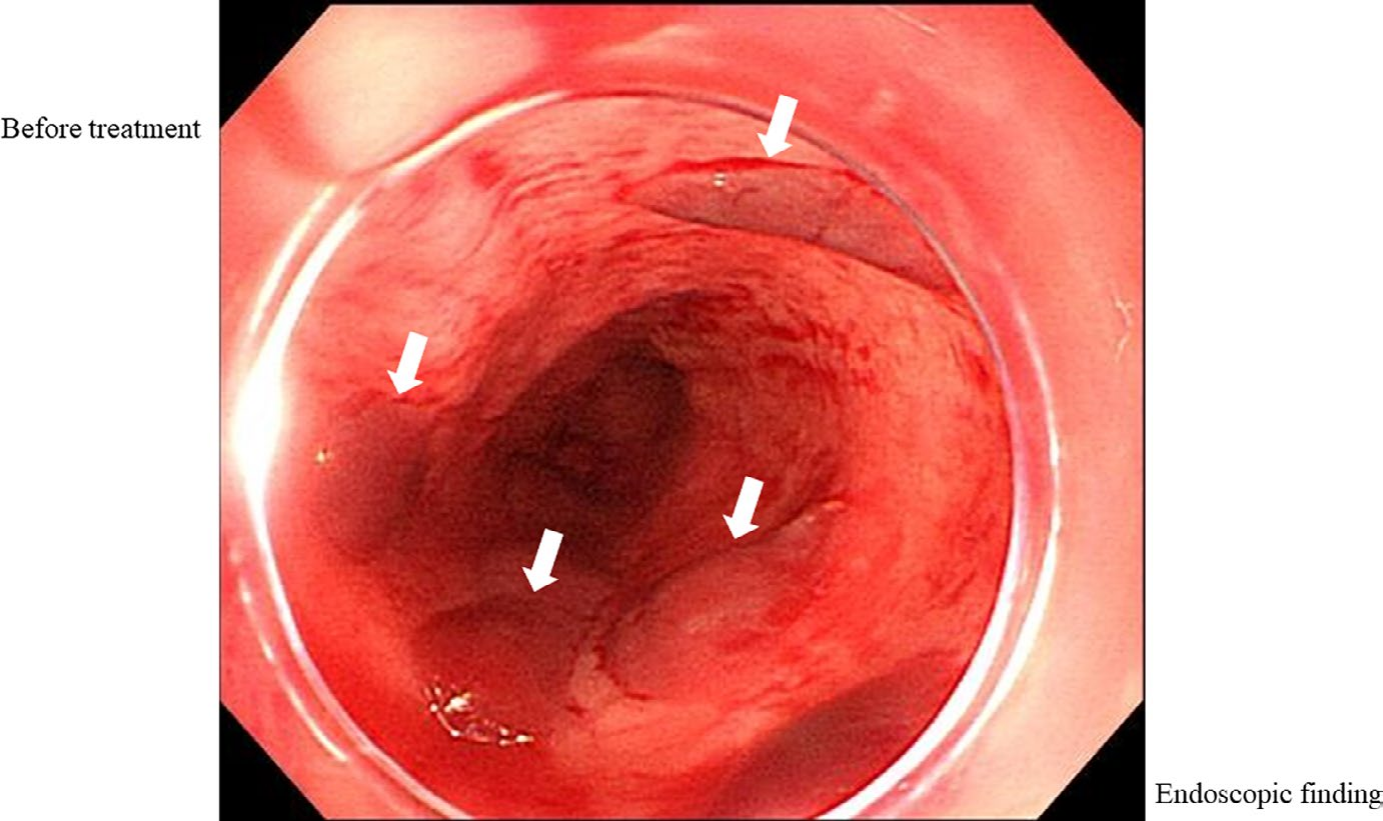
Upper gastrointestinal endoscopy shows hematomas (white arrows) in the upper esophagus

**FIGURE 3:**
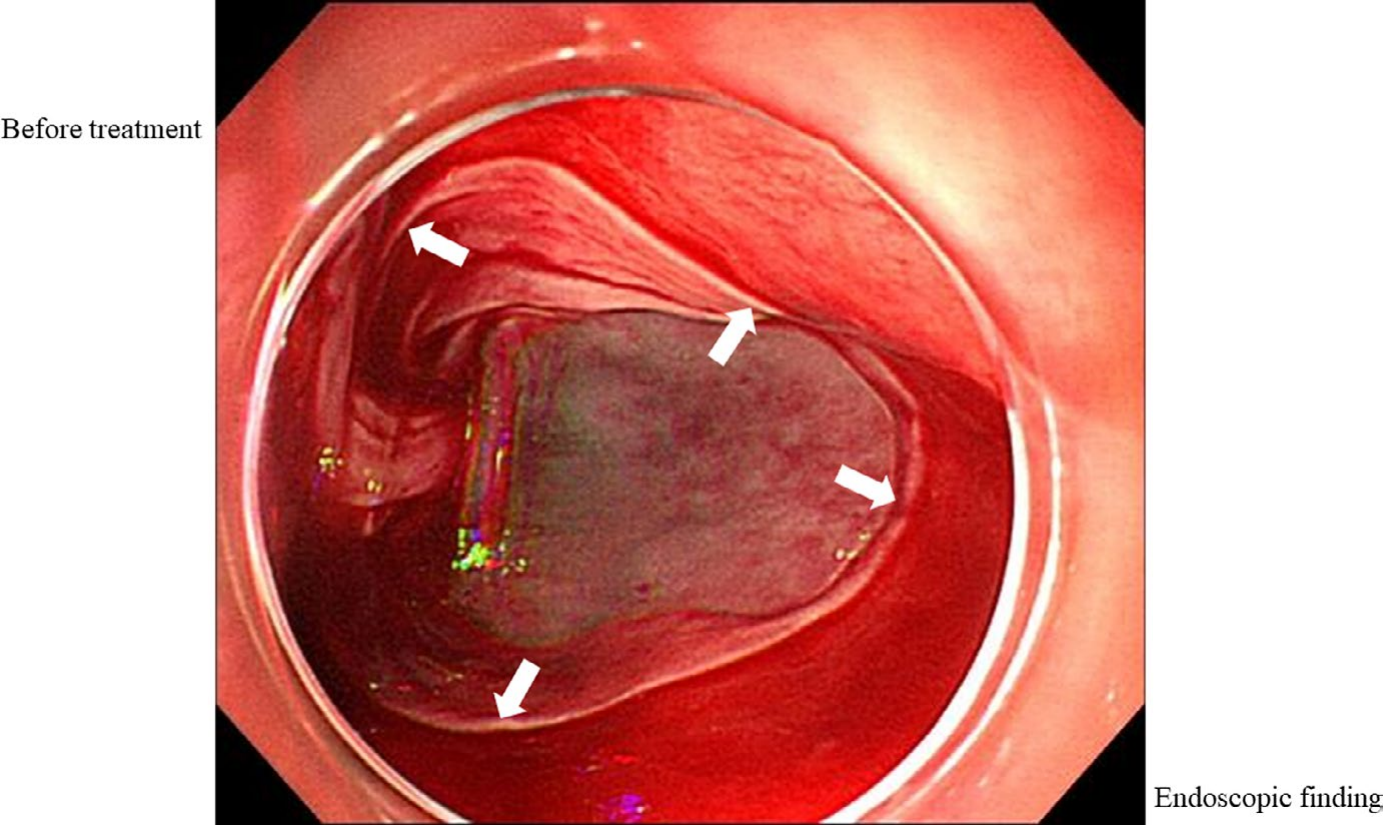
Upper gastrointestinal endoscopy shows peeled circumferential stratified squamous epithelium (white arrows) in the middle esophagus

A 73‐year‐old woman admitted with accidental swallowing of a foreign body. Upper gastrointestinal endoscopy showed a stuck press through package (PTP) in the pharyngoesophageal junction (Figure 1); however, during endoscopy, it passed through the esophagus and fell into the stomach by peristalsis. After passing the foreign body, we observed hematoma, peeled circumferential stratified squamous epithelium from the upper to middle esophagus, and oozing from erosive lesions (Figures 2 and 3). What do you think the underlying disease and the prognosis of this patient?

## 2 ANSWER

**FIGURE 4:**
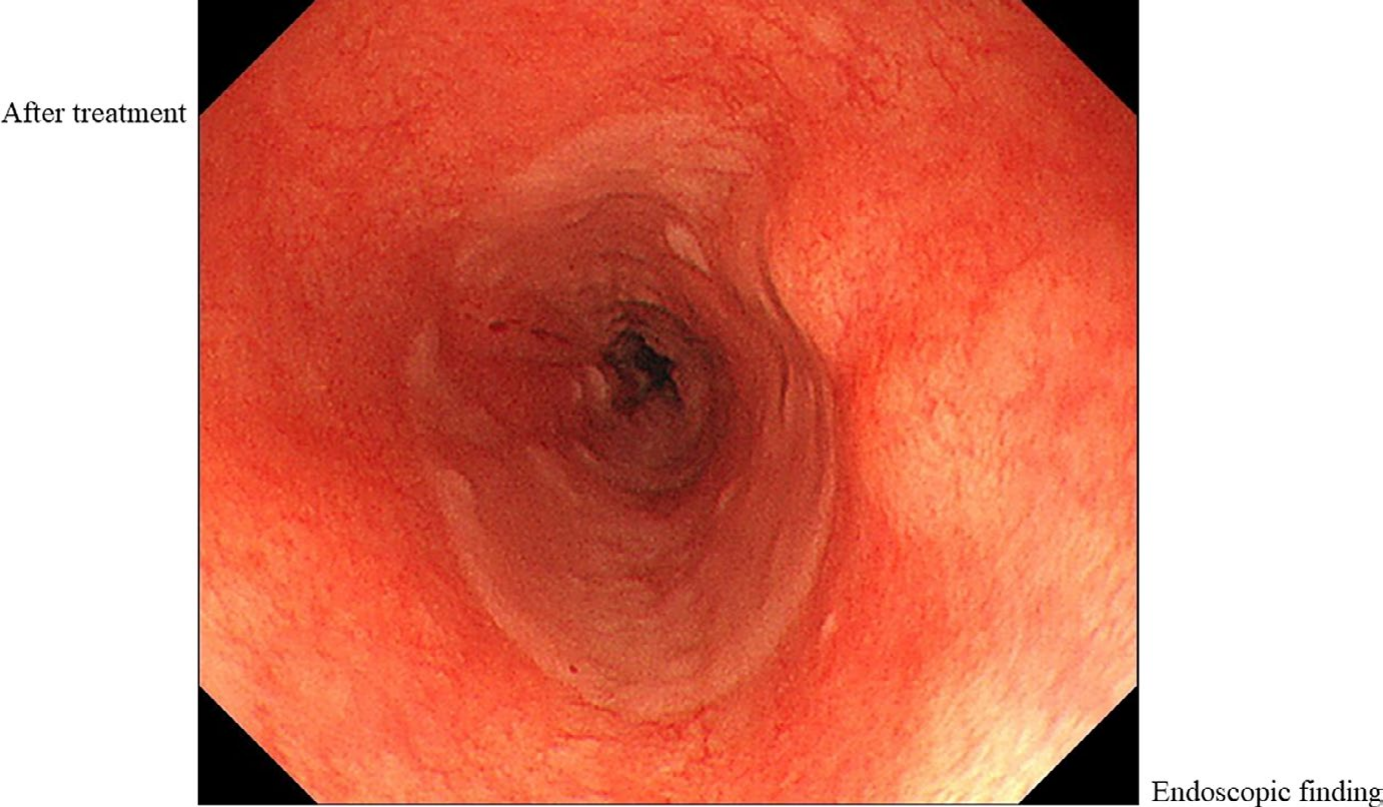
The esophageal epithelium was observed improvement tendency without stenosis by upper endoscopy

She was diagnosed with pemphigus vulgaris (PV). PV, the most common form of pemphigus, is a rare autoimmune bullous disease against desmoglein that shows acantholysis in the oral, pharyngeal, and esophageal stratified squamous epithelium.^1^, ^2^ However, there have been no reports of accidental foreign‐body swallowing. She was treated with proton‐pump inhibitors (PPI). The esophageal epithelium was observed improvement tendency without stenosis by endoscopy on day 3 after the event (Figure 4). The foreign body did not cause any adverse events. Therefore, she resumed eating and was discharged.

This report highlights the easy peeling of the esophageal epithelium with hematoma and oozing (Nikolsky phenomenon) after swallowing the foreign body and passing it into the esophagus and the healing status of the esophagus 3 days later. PPI treatment for several days during healing might be effective.

## CONFLICT OF INTEREST

The authors declare that they have no current financial arrangement or affiliation with any organization that may have a direct influence on their work. Informed consent was obtained from the patient for the publication of their information and imaging.

## AUTHOR CONTRIBUTION

All the authors made substantial contribution to the preparation of this manuscript and approved the final version for submission. KT, AT, HS: drafted the manuscript; AT: was the corresponding author; YI, NT, KN, YK, NK, YA, ST, KH, and JY: provided clinical support; ST: carefully reviewed the manuscript.
